# A Two-sample Nonparametric Test for Circular Data– its Exact Distribution and Performance

**DOI:** 10.1007/s13571-020-00244-9

**Published:** 2021-02-13

**Authors:** S. Rao Jammalamadaka, Stéphane Guerrier, Vasudevan Mangalam

**Affiliations:** 1 Department of Statistics & Applied Probability, University of California, Santa Barbara, USA; 2 Geneva School of Economics and Management, Faculty of Science, University of Geneva, Geneva, Switzerland; 3 School of Electrical Engineering, Computing and Mathematical Sciences, Curtin University, Perth, Australia

**Keywords:** Circular data, two-sample tests, spacing frequencies, small sample distributions, Wheeler-Watson, Dixon, Wilcoxon test, power., Primary 62G10, 62E15, Secondary 62Q05

## Abstract

A nonparametric test labelled ‘Rao Spacing-frequencies test’ is explored and developed for testing whether two circular samples come from the same population. Its exact distribution and performance relative to comparable tests such as the Wheeler-Watson test and the Dixon test in small samples, are discussed. Although this test statistic is shown to be asymptotically normal, as one would expect, this large sample distribution does not provide satisfactory approximations for small to moderate samples. Exact critical values for small samples are obtained and tables provided here, using combinatorial techniques, and asymptotic critical regions are assessed against these. For moderate sample sizes in-between i.e. when the samples are too large making combinatorial techniques computationally prohibitive but yet asymptotic regions do not provide a good approximation, we provide a simple Monte Carlo procedure that gives very accurate critical values. As is well-known, the large number of usual rank-based tests are not applicable in the context of circular data since the values of such ranks depend on the arbitrary choice of origin and the sense of rotation used (clockwise or anti-clockwise). Tests that are invariant under the group of rotations, depend on the data through the so-called ‘spacing frequencies’, the frequencies of one sample that fall in between the spacings (or gaps) made by the other. The Wheeler-Watson, Dixon, and the proposed Rao tests are of this form and are explicitly useful for circular data, but they also have the added advantage of being valid and useful for comparing any two samples on the real line. Our study and simulations establish the ‘Rao spacing-frequencies test’ as a desirable, and indeed preferable test in a wide variety of contexts for comparing two circular samples, and as a viable competitor even for data on the real line. Computational help for implementing any of these tests, is made available online “TwoCircles” R package and is part of this paper.

## Introduction

The primary goal of this paper is to explore and develop a test for comparing two circular samples, study its small sample distribution, and make it available to practitioners through tables of critical values, and an **R** code. Its small sample performance is assessed relative to comparable tests that are used in this context, and it is shown to be valid and superior in a broader context. When such samples are on the real line, there is a great deal of literature on tests based on the *ranks* of one of these samples in the combined sample, such as the Wilcoxon test (see e.g. the classical text by Hájek et al., 1999 or more recent texts such as Wasserman, 2006 or Gibbons and Chakraborti, 2011. However on a circle, ranks are not uniquely defined as their values depend on the choice of origin and the sense of rotation (i.e. clockwise/anticlockwise), which are both arbitrary. Discussions on circular data can be found, for example, in Rao and SenGupta (2001) and Mardia and Jupp (2009). An important alternative to rank tests is to consider tests based on the *spacing frequencies* which are the counts or the frequencies of one sample that fall in between the spacings or gaps made by the other sample. These frequencies remain unchanged under different choices of zero direction and the sense of rotation, and indeed, as the maximal invariant under the rotation group, they play a central role for testing problems that arise in connection with circular data (see e.g. Gatto and Rao, 2015).

The objective of this paper is to provide the exact distribution theory for a few important ones among these tests, namely the Wheeler-Watson test, the Dixon test and the newly introduced Rao test, provide their exact distribution theory and critical values, and evaluate their relative power performance in small samples. As two-sample tests for circular data come up widely in many physical and natural sciences (see e.g. Taylor and Burns, 2016), our results providing accurate critical values for such tests based on spacing-frequencies, have important practical implications when dealing with small and moderate sample sizes.

Although the main purpose of this new test lies in its use for comparing two circular samples, it is equally useful for comparing any two samples on the real line. Just to demonstrate this and to see how well it performs in that context, in Section 7 we make a power comparison with just one such test that is commonly used on the line, viz. the Wilcoxon test.

## Spacing-Frequencies Tests for Comparing two Circular Populations

A special note about the notations: while *m* observations on a circle make up an equal number of spacings, on the real line (*m* − 1) observations break up the real line into *m* spacings. So in order to accommodate this quirk, we start with (*m* − 1) observations for one of the samples as if they are on the line, and all the theory works equally well when there are *m* observations on the circle, which is our sample space of interest.

Let {*X*_*i*_, *i* = 1,…,*m* − 1} and {*Y*_*j*_, *j* = 1,…,*n*} be independent samples from two continuous distributions *F* and *G* respectively, on the real line. We want to test the classical null hypothesis *H*_0_ : *F* = *G* against suitable alternatives. On the real line, let *X*_(*i*)_, *i* = 1,…,*m* − 1 be the order statistics of the *X*_*i*_’s, with the notation $X_{(0)} \equiv -\infty \ \text {and} \ X_{(m)} \equiv \infty $. For *k* = 1,…,*m*, let *S*_*k*_ denote the number of *Y*_*j*_’s in the interval [*X*_(*k*− 1)_,*X*_(*k*)_), i.e. 
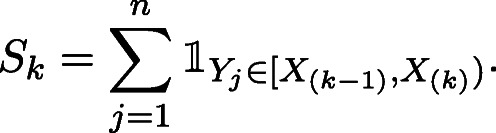


On the circle, one may start with any one of the observations as the “origin” and consider either sense of rotation (clockwise or anticlockwise) and define the frequency of *Y*_*j*_’s in the arc-lengths formed by the sample *X*_(*i*)_’s, *i* = 1,…,*m*. These *S*_*k*_’s are referred to as spacing frequencies, and their distribution theory remains the same.

Unlike the ranks which are not well-defined for circular data, such spacing frequencies have the rotational invariance property and play a prominent role in comparing two samples on the circle. They are equally useful on the line as they represent *rank-differences* (see Gatto and Rao, 2015 for details). Large sample theory for families of nonparametric tests of the form ${\sum }_{k=1}^{m} h(S_{k})$ which are symmetric in these spacing-frequencies as well as for the more general statistics of the form ${\sum }_{k} h_{k}(S_{k})$ are studied in Holst and Rao (1980). But as shown in Gatto and Rao (2015), symmetric statistics based on spacing-frequencies have the rotational invariance property and hence are appropriate in the circular context.

In this paper, we consider three such nonparametric tests based on spacing frequencies for comparing any two circular samples, and these are listed in Table 1 below.

**Table 1:** Test statistics based on *S*_*k*_’s

Name	Formula
Rao spacing-frequencies statistic	$T_{1}={\sum }_{k=1}^{m} \left |S_{k}-\frac {n}{m}\right |$
Dixon statistic	$T_{2}={\sum }_{k=1}^{m} (S_{k}-\frac {n}{m})^{2}$
Wheeler-Watson statistic	$T_{3} = \left \{ {\sum }_{k = 1}^{m} {\cos \limits } \left (\frac {2 \pi }{n+m}\left [k + {\sum }_{j = 1}^{k-1} S_{j}\right ]\right )\right \}^{2} +$
	$\phantom {T_{5} = } \left \{ {\sum }_{k = 1}^{m} {\sin \limits } \left (\frac {2 \pi }{n+m}\left [k + {\sum }_{j = 1}^{k-1} S_{j}\right ]\right )\right \}^{2}$

Note that, under the null hypothesis, the expected value of *S*_*k*_ is given by *n*/*m*. While the Dixon statistic (1940) looks at the squared *L*^2^ norm of $(S_{k}- \mathbb {E}[S_{k}])$ (which can be seen as also equivalent to $\sum {S_{k}^{2}}$), the Rao spacing-frequencies statistic given above, looks at the *L*^1^ norm of $(S_{k} - \mathbb {E}[S_{k}])$. As we show below, it is equal to the simple sum $2{\sum }_{k=1}^{m} \max \limits \left (S_{k}-\frac {n}{m},0\right )$. This is because

$$ \begin{aligned} \sum\limits_{k=1}^{m} \left| S_{k}-\frac{n}{m}\right| &= \sum\limits_{k = 1}^{m} \max\left( S_{k}-\frac{n}{m}, 0\right) + \sum\limits_{j = 1}^{m} \max\left( \frac{n}{m} - S_{j} , 0\right)\\ &= 2 \sum\limits_{k = 1}^{m} \max\left( S_{k}-\frac{n}{m}, 0\right), \end{aligned} $$

since

$$ 0 = \sum\limits_{k=1}^{m} \left( S_{k}-\frac{n}{m}\right) = \sum\limits_{k = 1}^{m} \max\left( S_{k}-\frac{n}{m}, 0\right) - \sum\limits_{j = 1}^{m} \max\left( \frac{n}{m} - S_{j} , 0\right). $$

This statistic may be seen as the two-sample analog of the frequently used “Rao’s spacings test” for testing uniformity or isotropy for a single sample of circular data (see Rao, 1976). The exact distribution theory, critical values, and the relative performance of this statistic *T*_1_ are being studied in some detail here for the first time. The Wheeler-Watson test (see Wheeler and Watson, 1964) is based on what are called *uniform scores* and can be written in the above form in terms of spacing frequencies.

### *Remark* 1.

One may construct tests of the same functional form based on the “dual” spacing-frequencies, namely the number of X-observations that fall in between the spacings made by the Y-observations, but as discussed in some detail in Gatto and Rao (2015) there is a one-to-one correspondence between these two sets, and one may proceed either way to obtain comparable conclusions. This duality may be seen as somewhat analogous to choosing the ranks of one of the two samples in the combined sample, in the theory of rank tests. Rao and Murthy (1981) consider a statistic based on the squared frequencies of both kinds and show that in large samples, this does not add to further efficiency compared to squaring just one set of frequencies as does the Dixon statistic mentioned below. Given this, the authors suggest taking the smaller of the two samples as the X’s to make up the spacings, and the larger sample as the Y’s for obtaining the frequencies in order to avoid many empty cells with zero frequencies (in analogy with having more balls than cells).

## Asymptotic Distributions

The asymptotic distributions of the Rao statistic, as well as the Dixon statistic, can be obtained using the general results of Holst and Rao (1980), in particular their Theorem 4.1, stated below.

### **Theorem 1** (Holst and Rao, 1980).

*Let T* *be a symmetric statistic of the form* ${\sum }_{k=1}^{m} h(S_{k})$. *Let η* *be a geometric* (*ρ*) *random variable where*

$$ \rho = \lim_{m,n\rightarrow\infty} \frac{m}{m+n} \in \left( 0, 1\right). $$

*Let*

1 $$ \mu= \mathbb{E}\left[h(\eta)\right] \ \ \ \text{and} \ \ \ \sigma^{2} = V\left[h(\eta)\right]-\frac{Cov^{2}\left[h(\eta),\eta\right]}{V\left[\eta\right]}. $$

*Then, under the null hypothesis H*_0_ *that the two populations are identical, the asymptotic distribution of T* *is given by*

2 $$ \frac{1}{\sqrt{m}\sigma}\sum\limits_{k=1}^{m} \left( T-m\mu\right)\xrightarrow{\mathcal{L}} \mathcal{N}(0,1). $$

**Figure 1:**
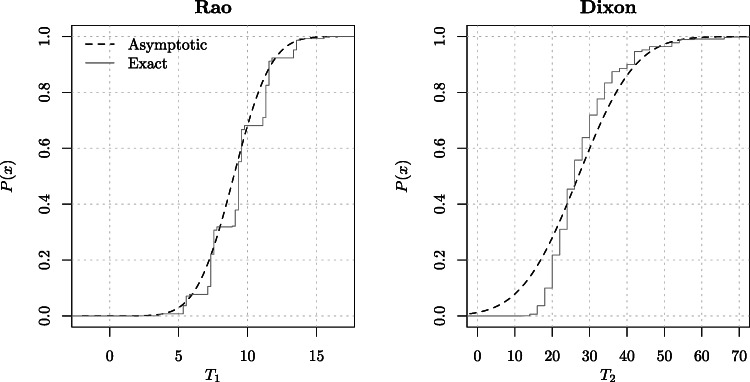
Exact and asymptotic distributions for the Rao and Dixon statistics for the case of *m* = 9 and *n* = 10

We conclude this section with the special case when the sample sizes from the two populations are equal, just in order to explore the divergence between this large sample result and the exact values, which we do in Fig. 1 later. In this case, Eq. 2 has a very simple expression for both these tests, as shown in the following corollary.

### **Corollary 1**.

*When the sample sizes from the two populations are equal, the limiting null distribution of the Rao statistic is given by*

$$ \frac{T_{1} - m}{\sqrt{m/2}} \xrightarrow[ ]{\mathcal{L}} \mathcal{N}(0,1). $$

*while the asymptotic distribution of the Dixon statistic is given by*

$$ \frac{T_{2} - 3m}{4 \sqrt{m}} \xrightarrow[ ]{\mathcal{L}} \mathcal{N}(0,1). $$

### *Proof*

From Eq. 1, when the sample sizes are equal, *η* denotes a random variable with a Geometric $\left (1/2\right )$ distribution, and therefore we have $\mathbb {E}[\eta ]=1$, $\mathbb {E}[\eta ^{2}]=3$, *V* [*η*] = 2, $\mathbb {E}[\eta ^{3}]=13$ and $\mathbb {E}[\eta ^{4}]=75$.

Thus for the Rao statistic with *h*(*x*) = |*x* − 1|, we can check that $\mathbb {E}\left [|\eta -1|\right ] = 1$. Moreover, we have $V[h(\eta )]= \mathbb {E}\left [(\eta -1)^{2}\right ]-\mathbb {E}^{2}[|\eta -1|] = 1$ and $Cov(h(\eta ),\eta ) = \mathbb {E}[\eta |\eta -1|]-1 = 1$.

Similarly for the Dixon statistic with *h*(*x*) = *x*^2^, therefore we have $V[h(\eta )]= V[\eta ^{2}] = \mathbb {E}[\eta ^{4}] - \mathbb {E}^{2}[\eta ^{2}] = 66$, and $Cov(h(\eta ),\eta ) =Cov(\eta ^{2},\eta ) = \mathbb {E}[\eta ^{3}]-\mathbb {E}[\eta ^{2}]\mathbb {E}[\eta ] = 10$, from which the result follows. □

## Exact Distributions

Mirakhmedov et al. (2014) provide improvements to the limiting normal approximation given in Theorem 1 through Edgeworth expansions. Gatto and Rao (1999) discuss the use of saddle-point approximations which lead to accurate numerical approximations that compare well with Monte Carlo simulations. However these are still attempts to get closer to the exact distributions, which is the primary focus of this paper.

The exact distributions of these test statistics can be found by considering the joint distribution of $\mathbf {S}=\left (S_{1}, S_{2}, \ldots , S_{m}\right )$. Since under the null hypothesis the *X*_*i*_’s and *Y*_*i*_’s come from the same distribution, all possible permutations of the *X*_*i*_’s and *Y*_*i*_’s are equally likely. Hence the distribution of **S** is found by looking at the number of ways *Y*_*i*_’s can be distributed among the spaces between the order statistics of *X*. Since there are *m* spaces generated by the *X*_(*k*)_’s and *n* objects are to be inserted into these *m* cells, which is the classical combinatorial problem, and can be done in $\binom {n+m-1}{m-1}$ equally likely ways. Hence each possible configuration of $\mathbf {S}=\left (S_{1}, S_{2}, \ldots , S_{m}\right )$ has probability $\binom {n+m-1}{m-1}^{-1}$. In order to derive the probability for any specific value of a given statistic, we need to add the probabilities of all combinations of **S** that correspond to this value. This involves increasingly complex combinatorial computations, for which we provide an **R** package.

### Computation of the Exact Critical Regions

We now consider the small sample case and provide the exact critical values for these test statistics, corresponding to commonly used significance levels of *α* = 0.10 and *α* = 0.05. Since these test statistics are all discrete, for any given significance level *α*, we sandwich such an *α* between upper tail probabilities *p*_1_ and *p*_2_ with the corresponding critical values *c*_1_ and *c*_2_ as follows. For each value of *α*, we give a pair of bracketing points (*c*_1_,*c*_2_) and corresponding upper tail probabilities (*p*_1_,*p*_2_), where *c*_1_ < *c*_2_ and *p*_1_ > *p*_2_. Using *c*_*i*_,*i* = 1,2 as the critical value and $[c_{i},\infty )$ as the critical region so that any observed value of *T* larger than or equal to *c*_*i*_ leads to *H*_0_ being rejected, we have a test of significance level *p*_*i*_,*i* = 1,2. The points (*c*_1_,*c*_2_) are chosen to be successive values of *T* so that *P*(*T* ∈ (*c*_1_,*c*_2_)|*H*_0_) = 0 and *p*_1_ = *P*(*T* ≥ *c*_1_|*H*_0_) ≤ *α*, *p*_2_ = *P*(*T* ≥ *c*_2_|*H*_0_) > *α*. The upper 10% and 5% critical values for the Rao, Dixon, and the Wheeler-Watson tests are given in Tables 3 to 5 in the Appendix, for small samples (values of *m* ≤ 12, *n* ≤ 11). For other choices of *m* and *n*, such critical values can be computed using the “TwoCircles” **R** package^2^ that we make available.

### Exact Versus Asymptotic Distributions

Such exact null distributions can now be compared to the asymptotic results given by Corollary 1. Figure 1 presents such a comparison for the Rao and Dixon statistics, taking for illustration *m* = 9 and *n* = 10,which are nearly equal size samples. This illustrates, as expected, that for small sample sizes the asymptotic distribution provides a very poor approximation to the exact one, especially for the Dixon statistic.

**Figure 2:**
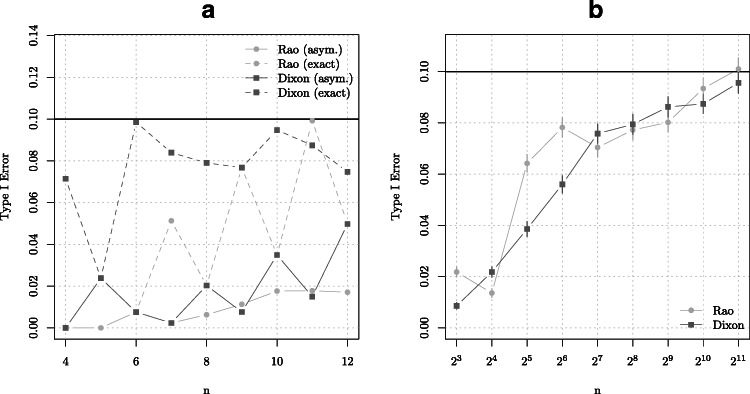
**a** Type I errors for the Dixon and Rao statistics obtained from the exact and asymptotic rejection regions. **b** The relationship between the sample size *n* and the type I error obtained from asymptotic rejection regions. The error bars correspond to the standard errors obtained by resampling techniques

We further illustrate the deficiency of using the asymptotic critical values instead of using the exact values obtained by the combinatorial methods. We do this by comparing the finite sample type I errors for the Dixon and Rao statistics obtained from the exact and the asymptotic rejection regions. These results are obtained by taking equal sample sizes (i.e. *n* = *m* − 1) with *n* = 2,3,…,12 and at level *α* = 0.1. The results are presented on the left panel of Fig. 2. As expected, the exact rejection regions lead to type I errors which are systematically closer to *α* than the ones obtained using asymptotic results. In the right panel of Fig. 2 we investigate by Monte Carlo simulations (under *H*_0_: $X_{i} \sim \mathcal {N}(0,1)$ and $Y_{i} \sim \mathcal {N}(0,1), i = 1,\ldots ,n$) the relationship between the sample size *n* and the empirical type I error obtained from asymptotic rejection regions. The results suggest that a sample size of a several thousands are needed to obtain a correct type I error. Overall conclusion is that the asymptotic distribution differs considerably from the exact distribution even for moderately large sample sizes. This calls for better approximations for dealing with moderate sample sizes, which is what we explore next.

## Critical Values for Moderate Sample Sizes via Simulation

For moderately large values of *m* and *n*, the calculation of the exact sampling distribution through exhaustive enumeration of all possible combinations may be too computationally intensive and, consequently, infeasible. However, these values may at the same time be too small for the asymptotic distribution to deliver a reliable approximation of the sampling distribution. Such cases may typically arise when ${\min \limits } (n, m) > 20$ and $\max \limits (n, m) < 1000$. To address this issue, we consider a simple and computationally efficient Monte Carlo approach to compute fairly accurate critical regions and p-values. This approach tends to reproduce the idea behind random permutations in general non-parametric context, while taking advantage of the known properties of the spacing-frequencies *S*_*k*_ under the null hypothesis.

This approach can be summarized in the following algorithm which is based on *B* Monte Carlo replications:

1. Let *b* = 1.
2. Draw the following two samples $\{X_{i}^{\ast }, \ \ i=1, \ldots , m-1\}$ and $\{Y_{j}^{\ast }, \ \ j=1, \ldots , n\}$ where $X_{i}^{\ast } \sim \mathcal {U}(0,1)$ and $Y_{j}^{\ast } \sim \mathcal {U}(0,1)$.
3. Compute the spacing-frequencies based on two samples obtained from the previous step, i.e. 
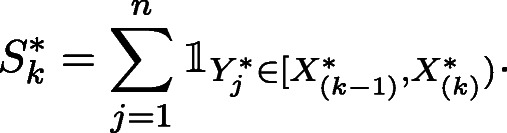
4. Compute the test statistic of interest based on the $S_{k}^{\ast }$’s previously obtained and define $T_{b}^{\ast }$ as follows:
  $$ T_{b}^{\ast} = \sum\limits_{k = 1}^{m} h(S_{k}^{\ast}). $$
5. If *b* < *B* go to step 2 and define *b* = *b* + 1, otherwise end the procedure.

As a result of this procedure, the empirical distribution $T_{1}^{\ast }, ..., T_{B}^{\ast }$ can be used to estimate the bracketing points ($c^{\ast }_{1},c^{\ast }_{2}$) together with the pair of significance levels ($p^{\ast }_{1},p^{\ast }_{2}$). Naturally, this approach can also be employed to calculate empirical p-values, which are simply defined as (see e.g. Davison and Hinkley, 1997) 
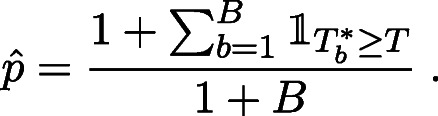


Moreover, the precision of the above approximations can be assessed using different techniques such as e.g. applying the standard non-parametric bootstrap on the*T*_*b*_’s, allowing to select a value of *B* that leads to the desired level of precision. The algorithm described in this section is implemented in the “TwoCircles” R package^3^ that we make available.

## Relative Performance of the Three Tests—Simulated Powers

Holst and Rao (1980) provide a comprehensive treatment on the asymptotic distribution theory and *asymptotic efficiencies* for tests based on spacing-frequencies. However, to calculate the *exact* distributions of these test statistics under alternatives, one needs the probability distribution of the vector **S** under the alternatives, which is quite complex and is given by the following

### *Remark* 2.

When *f* is the density of *X*, *G* the density of *Y*

$$ \begin{aligned} &P\left[S_{1}=n_{1},{\ldots} S_{m}=n_{m}\right] = \frac{(m-1)!n!}{n_{1}! n_{2}!{\ldots} n_{m}!}{\int}_{x_{1}<x_{2}<\ldots<x_{m-1}}G^{n_{1}}(x_{1}) \times \\ &\left( \prod\limits_{i=2}^{m-1} \left[G(x_{i})-G(x_{i-1})\right]^{n_{i}}\right) \left[1-G(x_{m-1})\right]^{n_{m}} \left( \prod\limits_{i=1}^{m-1} f(x_{i})\right)d\mathbf{x} , \end{aligned} $$

with *n*_1_ + *n*_2_ + ⋯*n*_*m*_ = *n*.

In view of this complexity, in this section, we investigate the finite sample performance of the Rao test, compared with the Dixon and the Wheeler-Watson tests via simulations, focusing on circular alternatives since these are primarily meant for circular situations. Different scenarios are considered, each corresponding to a different type of departure from the null hypothesis *H*_0_. Simulation 1 considers the case when the two samples come from two von Mises distributions with the same concentration, but as the difference in their mean directions increases.

### *Simulation* 1.

We consider the following setting:

$$ \begin{aligned} X_{i} &\sim \text{vM}(0,2), i =1,\ldots,m\\ Y_{j} &\sim \text{vM}(\mu,2), j =1,\ldots,n. \end{aligned} $$

**Figure 3:**
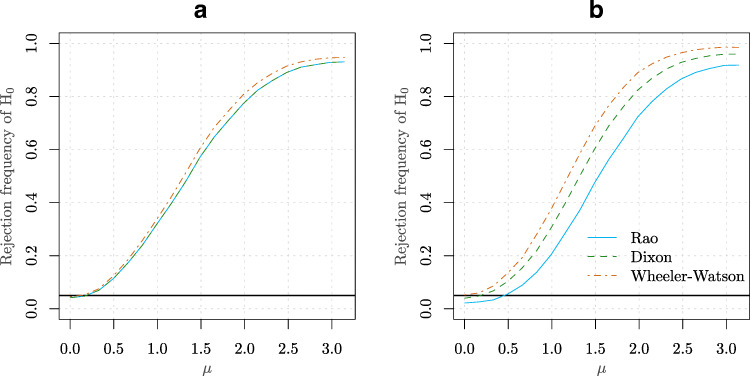
**a** Power curve for 20 equally spaced values of *μ* between 0 to *π* (see Simulation 1) for the Rao, Dixon, and Wheeler-Watson tests with *m* = 4 and *n* = 12. **b** Similar to Case **(a)** but for the values *m* = 8 and *n* = 8.

An equally spaced grid of 20 values for *μ* was employed ranging from 0 to *π*. Two combinations of sample sizes were used for *m* and *n*. The empirical power curves (based on 10^4^ Monte Carlo replications) of the different tests considered in Table 1 are presented in Fig. 3. It can be observed that the Wheeler-Watson test appears superior in this simulation to both the Rao and Dixon tests.

In the next simulation we consider again the case when the two samples come from two von Mises distributions with the same mean (at 0), but as the difference in their concentration increases.

### *Simulation* 2.

In this simulation we consider the following setting:

$$ \begin{aligned} X_{i} &\sim \text{vM} (0, 0), i =1,\ldots,m\\ Y_{j} &\sim \text{vM} (0, \delta), j =1,\ldots,n. \end{aligned} $$

**Figure 4:**
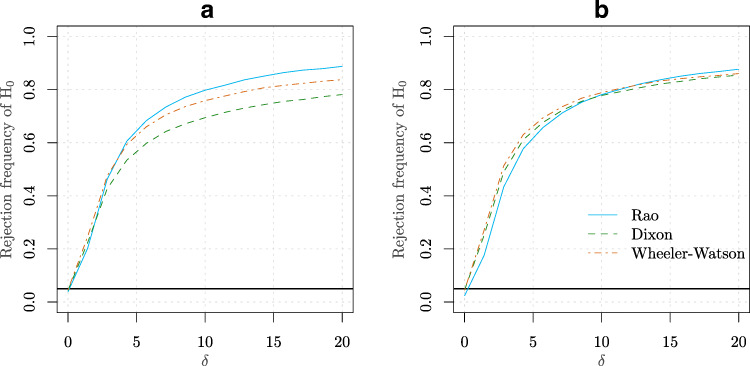
**a** Power curve for an equally spaced grid of 20 values for *δ* (see Simulation 2) ranging from 0 to 100 for the Rao, Dixon and Wheeler-Watson tests. The values *m* = 6 and *n* = 12 were considered in this case. **b** Similar to point **(a)** but for the values *m* = 6 and *n* = 16

An equally spaced grid of 20 values for *δ* was employed ranging from 0 to 20. Two combinations of sample sizes were used for *m* and *n*. The empirical power curves (based on 10^4^ Monte Carlo replications) are presented in Fig. 4. In this simulation, the Rao test appears to provide a better power than Wheeler-Watson and Dixon tests.

Next, we examine the performance of these tests when one of the samples is from a Uniform distribution on [0, 2*π*) (which is the same as a vM with zero concentration) while the other is from a mixture of two von Mises distributions.

### *Simulation* 3.

We consider the following setting:

$$ \begin{aligned} X_{i} &\sim \text{vM}(0,0), i =1,\ldots,m\\ Y_{j} &= \frac{1}{2} Y_{j}^{(1)} + \frac{1}{2} Y_{j}^{(2)} , j =1,\ldots,n\\ Y_{j}^{(1)} &\sim \text{vM}(\pi,\delta), Y_{j}^{(2)} \sim \text{vM}(0 ,\delta). \end{aligned} $$

**Figure 5:**
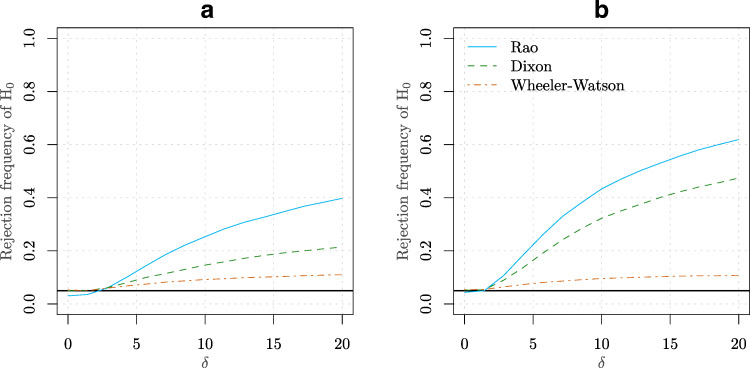
**a** Power curve based for an equally spaced grid of 20 values for *δ* (see Simulation 3) ranging from 0 to 20 for the Rao, Dixon and Wheeler-Watson tests. The values *m* = 6 and *n* = 22 were considered in this case. **b** Similar to point **(a)** but for the values *m* = 8 and *n* = 20

An equally spaced grid of 20 values for *δ* was employed ranging from 0 to 20. Two combinations of values were used for *m* and *n* such that the sum of the sample sizes was kept to 28. The empirical power curves (based on 10^4^ Monte Carlo replications) are presented in Fig. 5. Under this scenario, the Rao test clearly outperforms both the Wheeler-Watson and Dixon tests. One can observe that Wheeler-Watson test provides particularly poor performance in detecting departures from *H*_0_. This phenomenon is very similar to the one-sample situation where for testing uniformity, the Rayleigh test (which is based on the resultant length) fares poorly compared to the Rao’s Spacings test, when the samples come from a symmetric multimodal distribution, like axial data. See again Gatto and Rao (2015) for more details on this issue.

In the following simulation, we consider a different case of von Mises mixtures

### *Simulation* 4.

We consider the following setting:

$$ \begin{aligned} X_{i} &\sim \text{vM}(0 ,0), i =1,\ldots,m\\ Y_{j} &= \frac{1}{3} Y_{j}^{(1)} + \frac{1}{3} Y_{j}^{(2)} + \frac{1}{3} Y_{j}^{(3)}, j =1,\ldots,n\\ Y_{j}^{(1)} &\sim \text{vM}(0, \delta), Y_{j}^{(2)} \sim \text{vM}\left( \frac{2}{3}\pi, \delta\right), Y_{j}^{(3)} \sim \text{vM}\left( \frac{4}{3}\pi, \delta\right). \end{aligned} $$

**Figure 6:**
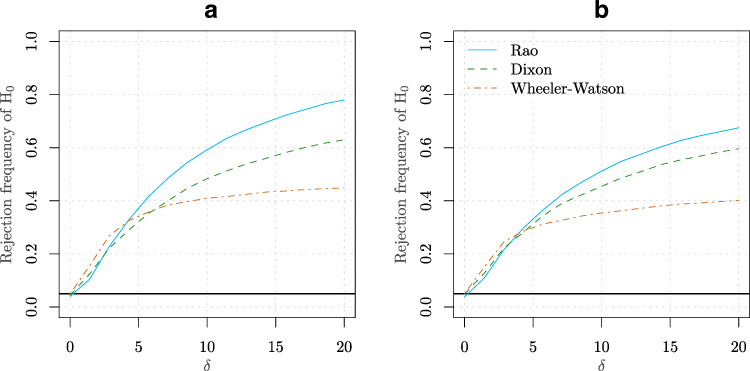
**a** Power curve based for an equally spaced grid of 20 values for *δ* (see Simulation 4) ranging from 0 to 20 for the Rao, Dixon and Wheeler-Watson tests. The values *m* = 10 and *n* = 18 were considered in this case. **b** Similar to point **(a)** but for the values *m* = 8 and *n* = 20

An equally spaced grid of 20 values for *δ* was employed ranging from 0 to 20. Two combinations of values were used for *m* and *n* such that the sum of the sample sizes was kept to 28. The empirical power curves (based on 10^4^ Monte Carlo replications) are presented in Fig. 6. Similar to the previous simulation, the Rao test clearly outperforms both the Wheeler-Watson and Dixon tests.

The examples presented here are but a sample of exhaustive small sample power study of these test statistics that we have undertaken. Based on these as well as the cases presented here, we can draw several useful conclusions. Among these 3 tests, the Wheeler-Watson test appears to perform better than the other two in detecting location shifts as indicated in Simulation 1. The Rao test appears preferable when one suspects that one of the samples may be from a mixture-distribution i.e. when one sample is transformed to be uniform on the circle, the other sample comes close to any symmetric bimodal or multimodal alternative such as a mixture of 2 von Mises distributions, as is the case in Simulations 3 and 4. This conclusion coincides with similar results obtained by Gatto and Rao (2015). The Dixon and Rao tests which are omnibus tests, perform well even in such situations. Although it is shown in Theorem 4.2 of Holst and Rao (1980) that the Dixon statistic is *asymptotically Locally Most Powerful* among symmetric functions of spacing-frequencies, we see that its performance in small samples is not as good as that of Rao test in all our simulations. The inevitable conclusion seems to be that one should use this Rao spacing-frequencies test if one suspects multimodal alternatives, or if the sample sizes are small to moderate - the case for which this paper provides tables and necessary **R** code.

## A Comparison with a Test on the Real Line

As stated earlier, let *X*_(*i*)_’s, *i* = 1,…,*m* − 1 denote the order statistics of *X*_*i*_ on the line with the notation $X_{(0)} \equiv -\infty $ and $X_{(m)}\equiv \infty $, and *S*_*k*_, *k* = 1,…,*m* denote the number of *Y*_*j*_’s in the interval [*X*_(*k*− 1)_,*X*_(*k*)_).

In order to make a simple comparison with two-sample nonparametric tests on the line, we consider one of the most commonly used tests namely the Wilcoxon test (see Wilcoxon (1945)) as a proxy. As shown in the lemma below, the Wilcoxon statistic is also a simple linear function of the spacing frequencies and belongs to this general class of tests. However it is not symmetric in these spacing-frequencies and hence takes different values depending on the choice of the origin. Because of this lack of rotational invariance, it cannot be used as such, for comparing two circular samples.

### **Lemma 1**.

*The Wilcoxon rank sum test statistic W* *can be written as:*

$$W=mn + \frac{m(m-1)}{2}-\sum\limits_{k=1}^{m} kS_{k},$$

in terms of {*S*_*k*_ : *k* = 1,…,*m*}.

### *Proof*

If *R*_*i*_ denotes the rank of *X*_(*i*)_ in the combined sample, the Wilcoxon statistic $W={\sum }_{i=1}^{m-1} R_{i}$. Since $R_{i}={\sum }_{k=1}^{i} (S_{k}+1)$, we have

$$ \begin{aligned} W=&\sum\limits_{i=1}^{m-1} R_{i}=\sum\limits_{i=1}^{m-1}\sum\limits_{k=1}^{i} (S_{k}+1) =\sum\limits_{k=1}^{m-1} (S_{k}+1)\sum\limits_{i=k}^{m-1}1 \\ =&\sum\limits_{k=1}^{m} (m-k)(S_{k}+1) = \sum\limits_{k=1}^{m} (m-k)S_{k} + \frac{m(m-1)}{2}. \end{aligned} $$

Since ${\sum }_{k=1}^{m} S_{k} = n$, we have ${\sum }_{k=1}^{m} (m-k)S_{k} = mn- {\sum }_{k=1}^{m} kS_{k}$ and the assertion follows. □

### *Remark* 3.

A centered version of *W* is sometimes used, namely the Mann-Whitney statistic $U=W-\frac {m(m-1)}{2}$, which can be written as

$$U=\sum\limits_{k=1}^{m} (m-k)S_{k} = mn-\sum\limits_{k=1}^{m} kS_{k}.$$

To briefly assess how the three tests considered in this paper fare with respect to competitors on the line, using the Wilcoxon test as a proxy, we consider a simple simulation, viz. Simulation 5.

### *Simulation* 5.

We consider the following setting:

$$ \begin{aligned} X_{i} &\sim \mathcal{N}(0,1), i =1,\ldots,m-1\\ Y_{j} &= p Y_{j}^{(1)} + (1 -p) \left( \frac{1}{2} Y_{j}^{(2)} + \frac{1}{2}Y_{j}^{(3)}\right), j =1,\ldots,n\\ Y_{j}^{(1)} &\sim \mathcal{N} (0, 1), Y_{j}^{(2)} \sim \mathcal{N} (\pi, 1), Y_{j}^{(3)} \sim \mathcal{N} (-\pi, 1). \end{aligned} $$

**Figure 7:**
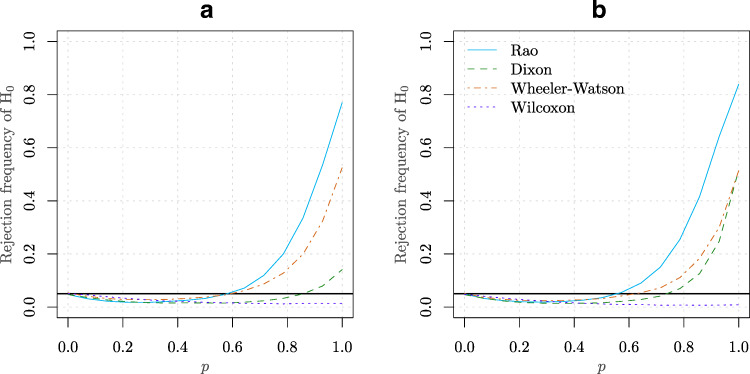
**a** Power curve for an equally spaced grid of 20 values for *p* (see Simulation 5) ranging from 0 to 1 for the tests considered in Table 1 as well as the Wilcoxon rank test. The values *m* = 5 and *n* = 18 were considered in this case. **b** Similar to point **(a)** but for the values *m* = 5 and *n* = 24

An equally spaced grid of 20 values for *p* was employed ranging from 0 to 1. Two combinations of values were used for *m* and *n*. The empirical power curves (based on 10^4^ Monte Carlo replications) of the tests considered in Table 1 as well as the Wilcoxon rank test, is presented in Fig. 7. Similar to the results presented in Section 6, Rao test clearly outperforms Wheeler-Watson, Dixon and even the Wilcoxon rank test. It can also be observed that the Wilcoxon rank test, and to a lesser extent the Wheeler-Watson test, provide a particularly poor performance in detecting departure from *H*_0_. This may be be partly explained by the fact that the distribution remains symmetric under the alternative.

## An Illustrative Example and Use of Tables

For small sample sizes (values of *m* ≤ 12, *n* ≤ 11), Appendix provides tables of critical values for these three test statistics, by giving bracketing values (closest critical value on either side) for *α* = 0.05,and *α* = 0.1. More extensive tables as well *p*-values can be obtained using R code available in the R package “TwoCircles”^4^. We illustrate the use of this code through the following example:


> library(TwoCircles)> get_critical_values(n = 6,m = 8, test = "rao", alpha = 0.05)Bracketing values (c1, c2) corresponding to significance levels(p1, p2) for Rao test based on the significance level 0.05c1 = 9 (p1 = 0.0862)c2 = 10.5 (p2 = 0.0047)

**Figure 8:**
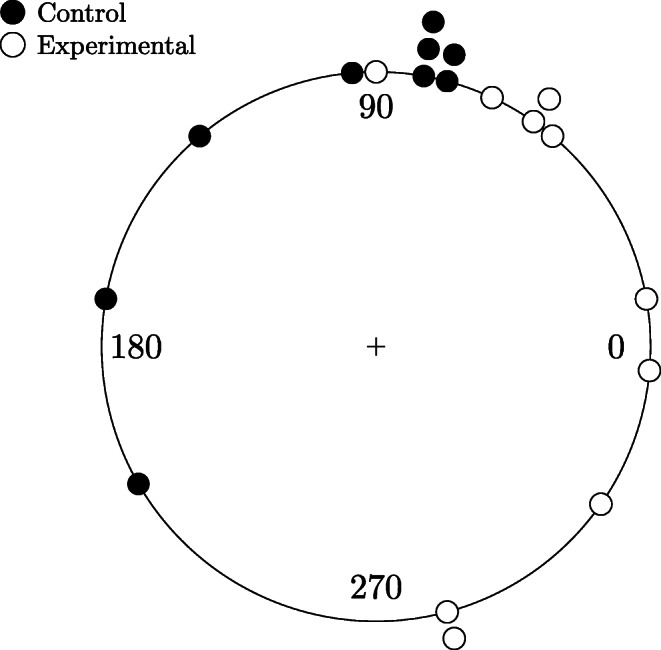
Two sample from the experiment on the homing orientation of pigeons. The reported angles in degrees are: 75°, 75°, 80°, 80°, 80°, 95°, 130°, 170°, 210° (control group); 10°, 50°, 55°, 55°, 65°, 90°, 285°, 285°, 325°, 355° (experimental group). Adapted from Figure 22 of Schmidt-Koenig (1958)

We now consider how these circular tests perform and their exact and asymptotic *p*-values, by applying them on a classic data set on homing pigeons. Homing pigeons are selectively bred pigeons that possess the ability to find their way home over extremely long distances, and are used in experiments on animal navigation. There is considerable literature on this subject (see e.g. Walcott, 1996 and the references therein). Among the early studies on homing pigeons, Schmidt-Koenig (1958) evaluated the difference in orientation abilities of a group of *control* and *experimental* birds. The experimental birds had their internal clock reset by six hours clockwise and were expected to deviate by about 90° counterclockwise with respect to the control birds upon release (see Schmidt-Koenig, 1958 for details). The release flight direction of the pigeons from the two groups were collected in different experiments and one of them is presented in Fig. 8. It can be observed that mean directions of the two groups appear to significantly differ. The experimental and control birds have a mean direction of respectively 18.9° and 104.0°, which is close to 90° (counterclockwise) expected difference.

A hypothesis of interest here is whether the observed directions of the two groups are from the same circular distribution. The three tests discussed in Section 2 can be used to perform such analysis. Their asymptotic and exact *p*-values are reported in Table 2. These results can be replicated using our package as illustrated through the following example:


> library(TwoCircles)> data("pigeons")> circular_test(pigeons$control, pigeons$experimental, test = "rao") Rao Two Sample TestData: pigeons$control and pigeons$experimentalTest Statistic: 15.77778Exact P-value: 0.00185Bracketing Points and Pair of Signif. Levels:c1 = 13.3333 (p1 = 0.0767)c2 = 13.5556 (p2 = 0.0479)

**Table 2:** Test statistics (Dixon, Rao and Wheeler-Watson), bracketing points (for *α* = 0.05) as well as asymptotic and exact *p*-values for the pigeons homing experiment

		Bracketing Points		Pair of Signif. Levels		P-Value	
Test	Statistic	*c*_1_	*c*_2_	*p*_1_	*p*_2_	Asymptotic	Exact
Dixon	82.00	44.00	46.00	0.0537	0.0479	0.1216	0.0019
Rao	15.78	13.33	13.56	0.0767	0.0479	0.0034	0.0019
Wheeler-Watson	25.72	14.58	14.61	0.0500	0.0498	0.0295	0.0039

The differences between the asymptotic and exact *p*-values are noticeable, in particular for the Dixon test where the asymptotic and exact *p*-values are 12.16% and 0.19%, respectively. In such case, one would actually be likely not to reject the null hypothesis simply due to the poor approximation of the asymptotic distribution for such a small sample size.

For the sample sizes of *m* = 9 and *n* = 10 as in this example, Fig. 1 can be used to read off the asymptotic and exact *p*-values for the Dixon and Rao tests. The poor asymptotic approximation in the case of Dixon test is particularly apparent from this figure, which confirms the large difference reported in Table 2.

## Concluding Remarks

A straightforward combinatorial approach is used to derive the exact distributions of several tests based on spacing frequencies. This class of tests for comparing two circular samples includes the newly introduced Rao test, the Dixon test, and the Wheeler-Watson test. For all these statistics our method provides the exact critical values and tables. Although the asymptotic theory of these statistics has been well-studied, such results often lead to a poor approximation in small to moderate samples, as we demonstrate. The comparative power performance of these various tests in small samples has been studied through extensive simulations, some of which are presented here. Based on these simulations, we find that omnibus tests like the Rao test and the Dixon test are preferable to the Wheeler-Watson test if one suspects symmetric multimodal alternatives. Again in small to moderate samples, Rao spacing frequencies test often outperforms the Dixon test. An illustrative comparison with the Wilcoxon test which is commonly used on the real line for comparing two samples, is presented to demonstrate that these tests may also be effectively used in two-sample comparisons on the real line.

## Appendix A. Tables of Critical Values

**Table 3:** Bracketing values (*c*_1_,*c*_2_) corresponding to significance levels (*p*_1_,*p*_2_)

		*α* = 0.10		*α* = 0.05	
*n*	*m*	*c*_1_(*p*_1_)	*c*_2_(*p*_2_)	*c*_1_(*p*_1_)	*c*_2_(*p*_2_)
5	6	6.67 (0.14)	8.33 (0.02)	6.67 (0.14)	8.33 (0.02)
5	7	7.14 (0.20)	8.57 (0.02)	7.14 (0.20)	8.57 (0.02)
5	8	7.50 (0.15)	8.75 (0.01)	7.50 (0.15)	8.75 (0.01)
5	9	7.78 (0.12)	8.89 (0.01)	7.78 (0.12)	8.89 (0.01)
5	10	7.00 (0.45)	8.00 (0.09)	8.00 (0.09)	9.00 (0.00)
5	11	7.27 (0.41)	8.18 (0.08)	8.18 (0.08)	9.09 (0.00)
5	12	7.50 (0.37)	8.33 (0.06)	8.33 (0.06)	9.17 (0.00)
6	6	8.00 (0.18)	10.00 (0.01)	8.00 (0.18)	10.00 (0.01)
6	7	8.57 (0.12)	10.29 (0.01)	8.57 (0.12)	10.29 (0.01)
6	8	7.50 (0.41)	9.00 (0.09)	9.00 (0.09)	10.50 (0.00)
6	9	8.00 (0.34)	9.33 (0.06)	9.33 (0.06)	10.67 (0.00)
6	10	8.40 (0.29)	9.60 (0.05)	8.40 (0.29)	9.60 (0.05)
6	11	8.73 (0.24)	9.82 (0.04)	8.73 (0.24)	9.82 (0.04)
6	12	9.00 (0.21)	10.00 (0.03)	9.00 (0.21)	10.00 (0.03)
7	6	9.33 (0.12)	9.67 (0.05)	9.33 (0.12)	9.67 (0.05)
7	7	8.00 (0.38)	10.00 (0.08)	10.00 (0.08)	12.00 (0.00)
7	8	8.75 (0.30)	10.50 (0.05)	10.50 (0.05)	12.25 (0.00)
7	9	9.33 (0.23)	10.89 (0.03)	9.33 (0.23)	10.89 (0.03)
7	10	9.80 (0.18)	11.20 (0.02)	9.80 (0.18)	11.20 (0.02)
7	11	10.18 (0.14)	11.45 (0.02)	10.18 (0.14)	11.45 (0.02)
7	12	10.50 (0.12)	11.67 (0.01)	10.50 (0.12)	11.67 (0.01)
8	6	9.33 (0.13)	10.67 (0.09)	10.67 (0.09)	11.33 (0.03)
8	7	9.43 (0.23)	9.71 (0.09)	11.43 (0.05)	11.71 (0.02)
8	8	10.00 (0.21)	12.00 (0.03)	10.00 (0.21)	12.00 (0.03)
8	9	10.67 (0.16)	12.44 (0.02)	10.67 (0.16)	12.44 (0.02)
8	10	11.20 (0.12)	12.80 (0.01)	11.20 (0.12)	12.80 (0.01)
8	11	10.18 (0.35)	11.64 (0.09)	11.64 (0.09)	13.09 (0.01)
8	12	10.67 (0.30)	12.00 (0.07)	12.00 (0.07)	13.33 (0.01)
9	6	10.00 (0.24)	11.00 (0.09)	12.00 (0.06)	13.00 (0.02)
9	7	10.86 (0.16)	11.43 (0.06)	11.43 (0.06)	12.86 (0.03)
9	8	11.50 (0.11)	11.75 (0.03)	11.50 (0.11)	11.75 (0.03)
9	9	12.00 (0.11)	14.00 (0.01)	12.00 (0.11)	14.00 (0.01)
9	10	10.80 (0.30)	12.60 (0.08)	12.60 (0.08)	14.40 (0.01)
9	11	11.45 (0.25)	13.09 (0.05)	13.09 (0.05)	14.73 (0.00)
9	12	12.00 (0.20)	13.50 (0.04)	12.00 (0.20)	13.50 (0.04)
10	6	11.33 (0.15)	12.67 (0.07)	12.67 (0.07)	13.33 (0.05)
10	7	12.29 (0.12)	13.14 (0.04)	12.29 (0.12)	13.14 (0.04)
10	8	12.50 (0.12)	13.00 (0.07)	13.00 (0.07)	13.50 (0.02)
10	9	11.56 (0.12)	11.78 (0.09)	13.33 (0.08)	13.56 (0.05)
10	10	12.00 (0.24)	14.00 (0.05)	14.00 (0.05)	16.00 (0.00)
10	11	12.73 (0.18)	14.55 (0.03)	12.73 (0.18)	14.55 (0.03)
10	12	13.33 (0.13)	15.00 (0.02)	13.33 (0.13)	15.00 (0.02)
11	6	12.67 (0.15)	14.33 (0.05)	12.67 (0.09)	14.33 (0.05)
11	7	12.57 (0.16)	12.86 (0.10)	13.71 (0.09)	14.86 (0.03)
11	8	12.50 (0.18)	13.25 (0.10)	14.50 (0.05)	15.25 (0.01)
11	9	13.11 (0.12)	13.56 (0.06)	14.67 (0.05)	15.11 (0.03)
11	10	13.40 (0.16)	13.60 (0.08)	13.60 (0.08)	13.80 (0.04)
11	11	14.00 (0.13)	16.00 (0.02)	14.00 (0.13)	16.00 (0.02)
11	12	12.83 (0.28)	14.67 (0.10)	14.67 (0.10)	16.50 (0.01)

**Rao Test** (*m* = number of spacings; *n* = total of frequencies)

**Table 4:** Bracketing values (*c*_1_,*c*_2_) corresponding to significance levels (*p*_1_,*p*_2_)

		*α* = 0.10		*α* = 0.05	
*n*	*m*	*c*_1_(*p*_1_)	*c*_2_(*p*_2_)	*c*_1_(*p*_1_)	*c*_2_(*p*_2_)
5	6	17.00 (0.14)	25.00 (0.02)	17.00 (0.14)	25.00 (0.02)
5	7	17.00 (0.11)	25.00 (0.02)	17.00 (0.11)	25.00 (0.02)
5	8	13.00 (0.15)	17.00 (0.08)	17.00 (0.08)	25.00 (0.01)
5	9	13.00 (0.12)	17.00 (0.06)	17.00 (0.06)	25.00 (0.01)
5	10	11.00 (0.27)	13.00 (0.09)	13.00 (0.09)	17.00 (0.05)
5	11	11.00 (0.24)	13.00 (0.08)	13.00 (0.08)	17.00 (0.04)
5	12	11.00 (0.21)	13.00 (0.06)	13.00 (0.06)	17.00 (0.03)
6	6	20.00 (0.14)	26.00 (0.08)	26.00 (0.08)	36.00 (0.01)
6	7	18.00 (0.23)	20.00 (0.10)	26.00 (0.05)	36.00 (0.01)
6	8	18.00 (0.18)	20.00 (0.07)	20.00 (0.07)	26.00 (0.04)
6	9	18.00 (0.15)	20.00 (0.05)	20.00 (0.05)	26.00 (0.03)
6	10	18.00 (0.12)	20.00 (0.04)	18.00 (0.12)	20.00 (0.04)
6	11	14.00 (0.22)	18.00 (0.10)	18.00 (0.10)	20.00 (0.03)
6	12	14.00 (0.19)	18.00 (0.08)	18.00 (0.08)	20.00 (0.02)
7	6	27.00 (0.16)	29.00 (0.08)	29.00 (0.08)	37.00 (0.05)
7	7	27.00 (0.11)	29.00 (0.05)	29.00 (0.05)	37.00 (0.03)
7	8	25.00 (0.10)	27.00 (0.08)	27.00 (0.08)	29.00 (0.03)
7	9	21.00 (0.15)	25.00 (0.07)	27.00 (0.06)	29.00 (0.02)
7	10	21.00 (0.12)	25.00 (0.06)	25.00 (0.06)	27.00 (0.05)
7	11	19.00 (0.19)	21.00 (0.09)	21.00 (0.09)	25.00 (0.04)
7	12	19.00 (0.16)	21.00 (0.08)	21.00 (0.08)	25.00 (0.03)
8	6	34.00 (0.12)	38.00 (0.10)	40.00 (0.05)	50.00 (0.03)
8	7	30.00 (0.16)	32.00 (0.09)	38.00 (0.07)	40.00 (0.03)
8	8	30.00 (0.11)	32.00 (0.06)	34.00 (0.05)	38.00 (0.04)
8	9	28.00 (0.12)	30.00 (0.08)	30.00 (0.08)	32.00 (0.04)
8	10	26.00 (0.12)	28.00 (0.09)	30.00 (0.06)	32.00 (0.03)
8	11	24.00 (0.11)	26.00 (0.10)	28.00 (0.07)	30.00 (0.04)
8	12	22.00 (0.17)	24.00 (0.08)	28.00 (0.06)	30.00 (0.03)
9	6	41.00 (0.15)	45.00 (0.08)	51.00 (0.06)	53.00 (0.03)
9	7	39.00 (0.13)	41.00 (0.10)	41.00 (0.10)	45.00 (0.05)
9	8	35.00 (0.12)	39.00 (0.09)	41.00 (0.06)	45.00 (0.03)
9	9	33.00 (0.11)	35.00 (0.08)	39.00 (0.06)	41.00 (0.04)
9	10	31.00 (0.13)	33.00 (0.08)	35.00 (0.06)	39.00 (0.05)
9	11	29.00 (0.14)	31.00 (0.10)	33.00 (0.06)	35.00 (0.05)
9	12	29.00 (0.11)	31.00 (0.08)	31.00 (0.08)	33.00 (0.04)
10	6	52.00 (0.12)	54.00 (0.09)	58.00 (0.05)	66.00 (0.04)
10	7	46.00 (0.11)	50.00 (0.08)	54.00 (0.06)	58.00 (0.03)
10	8	42.00 (0.14)	44.00 (0.08)	52.00 (0.05)	54.00 (0.03)
10	9	40.00 (0.11)	42.00 (0.10)	44.00 (0.05)	46.00 (0.05)
10	10	36.00 (0.12)	38.00 (0.09)	42.00 (0.07)	44.00 (0.04)
10	11	34.00 (0.13)	36.00 (0.09)	42.00 (0.05)	44.00 (0.03)
10	12	32.00 (0.14)	34.00 (0.10)	40.00 (0.05)	42.00 (0.04)
11	6	59.00 (0.12)	61.00 (0.09)	69.00 (0.06)	73.00 (0.04)
11	7	55.00 (0.11)	57.00 (0.08)	65.00 (0.05)	67.00 (0.05)
11	8	51.00 (0.10)	53.00 (0.10)	55.00 (0.08)	57.00 (0.05)
11	9	45.00 (0.12)	47.00 (0.10)	55.00 (0.05)	57.00 (0.03)
11	10	43.00 (0.14)	45.00 (0.09)	49.00 (0.05)	51.00 (0.05)
11	11	43.00 (0.10)	45.00 (0.06)	45.00 (0.06)	47.00 (0.05)
11	12	39.00 (0.10)	41.00 (0.09)	43.00 (0.08)	45.00 (0.05)

**Dixon Test** (*m* = number of spacings; *n* = total of frequencies)

**Table 5:** Bracketing values (*c*_1_,*c*_2_) corresponding to significance levels (*p*_1_,*p*_2_)

		*α* = 0.10		*α* = 0.05	
*n*	*m*	*c*_1_(*p*_1_)	*c*_2_(*p*_2_)	*c*_1_(*p*_1_)	*c*_2_(*p*_2_)
5	6	5.99 (0.12)	6.60 (0.10)	8.74 (0.06)	12.34 (0.02)
5	7	5.73 (0.12)	7.46 (0.10)	8.46 (0.05)	10.46 (0.05)
5	8	7.33 (0.10)	7.39 (0.10)	10.15 (0.05)	12.00 (0.03)
5	9	7.85 (0.10)	8.85 (0.08)	10.30 (0.05)	10.54 (0.04)
5	10	7.91 (0.10)	8.40 (0.09)	10.25 (0.05)	10.36 (0.05)
5	11	8.11 (0.10)	9.03 (0.09)	10.44 (0.05)	11.52 (0.04)
5	12	8.25 (0.10)	8.76 (0.10)	11.03 (0.05)	11.17 (0.05)
6	6	7.46 (0.11)	8.00 (0.10)	8.46 (0.06)	11.20 (0.04)
6	7	7.59 (0.11)	7.94 (0.10)	10.42 (0.05)	10.86 (0.04)
6	8	7.94 (0.10)	9.30 (0.09)	10.10 (0.05)	12.21 (0.04)
6	9	9.12 (0.10)	9.42 (0.09)	11.36 (0.05)	11.61 (0.04)
6	10	9.11 (0.10)	9.44 (0.09)	11.29 (0.05)	11.54 (0.04)
6	11	9.41 (0.10)	9.53 (0.10)	12.21 (0.05)	12.36 (0.05)
6	12	9.94 (0.10)	10.11 (0.09)	12.17 (0.05)	12.25 (0.05)
7	6	7.59 (0.12)	7.94 (0.10)	10.42 (0.05)	10.86 (0.04)
7	7	8.85 (0.10)	8.94 (0.09)	10.54 (0.05)	11.65 (0.05)
7	8	8.99 (0.10)	9.06 (0.10)	11.53 (0.05)	11.57 (0.05)
7	9	9.78 (0.10)	10.11 (0.09)	12.23 (0.05)	12.66 (0.05)
7	10	10.02 (0.10)	10.07 (0.10)	12.58 (0.05)	12.63 (0.05)
7	11	10.29 (0.10)	10.41 (0.09)	13.41 (0.05)	13.45 (0.05)
7	12	10.48 (0.10)	10.55 (0.10)	13.54 (0.05)	13.61 (0.05)
8	6	7.94 (0.10)	9.30 (0.09)	10.10 (0.05)	12.21 (0.04)
8	7	8.99 (0.10)	9.06 (0.10)	10.95 (0.05)	11.53 (0.05)
8	8	9.44 (0.10)	10.01 (0.10)	11.99 (0.05)	13.14 (0.05)
8	9	10.15 (0.10)	10.18 (0.10)	13.01 (0.05)	13.25 (0.05)
8	10	10.60 (0.10)	10.92 (0.10)	13.82 (0.05)	13.99 (0.05)
8	11	11.08 (0.10)	11.12 (0.10)	14.29 (0.05)	14.29 (0.05)
8	12	11.47 (0.10)	11.49 (0.10)	14.66 (0.05)	14.77 (0.05)
9	6	9.12 (0.10)	9.42 (0.09)	11.36 (0.05)	11.61 (0.04)
9	7	9.24 (0.10)	9.78 (0.10)	11.64 (0.05)	12.23 (0.05)
9	8	10.15 (0.10)	10.18 (0.10)	13.25 (0.05)	13.36 (0.05)
9	9	10.61 (0.10)	10.92 (0.10)	13.82 (0.05)	13.99 (0.05)
9	10	11.37 (0.10)	11.40 (0.10)	14.58 (0.05)	14.61 (0.05)
9	11	11.93 (0.10)	11.97 (0.10)	15.45 (0.05)	15.47 (0.05)
9	12	12.34 (0.10)	12.35 (0.10)	15.80 (0.05)	15.85 (0.05)
10	6	9.11 (0.10)	9.44 (0.09)	11.29 (0.05)	11.54 (0.04)
10	7	10.07 (0.10)	10.11 (0.10)	12.58 (0.05)	12.63 (0.05)
10	8	10.60 (0.10)	10.92 (0.10)	13.82 (0.05)	13.99 (0.05)
10	9	11.37 (0.10)	11.40 (0.10)	14.58 (0.05)	14.61 (0.05)
10	10	12.07 (0.10)	12.19 (0.10)	15.28 (0.05)	15.39 (0.05)
10	11	12.58 (0.10)	12.59 (0.10)	16.06 (0.05)	16.08 (0.05)
10	12	13.05 (0.10)	13.07 (0.10)	16.73 (0.05)	16.76 (0.05)
11	6	9.41 (0.10)	9.53 (0.10)	12.21 (0.05)	12.36 (0.05)
11	7	10.29 (0.10)	10.41 (0.09)	13.41 (0.05)	13.45 (0.05)
11	8	11.08 (0.10)	11.12 (0.10)	14.28 (0.05)	14.29 (0.05)
11	9	11.93 (0.10)	11.97 (0.10)	15.45 (0.05)	15.47 (0.05)
11	10	12.58 (0.10)	12.59 (0.10)	16.06 (0.05)	16.08 (0.05)
11	11	13.19 (0.10)	13.23 (0.10)	16.93 (0.05)	16.96 (0.05)
11	12	13.72 (0.10)	13.72 (0.10)	17.54 (0.05)	17.56 (0.05)

**Wheeler-Watson Test** (*m* = number of spacings; *n* = total of frequencies)

## Footnotes

2 available on GitHub: https://github.com/SMAC-Group/TwoCircles

3 available on GitHub: https://github.com/SMAC-Group/TwoCircles

4 available on GitHub: https://github.com/SMAC-Group/TwoCircles.
